# A small fishing vessel recognition method using transfer learning based on laser sensors

**DOI:** 10.1038/s41598-023-31319-y

**Published:** 2023-04-12

**Authors:** Jianli Zheng, Jianjun Cao, Kun Yuan, Yang Liu

**Affiliations:** 1 Fishery Machinery and Instrument Research Institute, Chinese Academy of Fishery Sciences, Shanghai, 201606 China; 2 School of Computer and Information Technology, Liaoning Normal University, Dalian, 116081 Liaoning China

**Keywords:** Computational science, Computer science, Information technology, Ocean sciences

## Abstract

The management of small vessels has always been key to maritime administration. This paper presents a novel method for recognizing small fishing vessels based on laser sensors. Using four types of small fishing vessels as targets, a recognition method for small fishing vessels based on Markov transition field (MTF) time-series images and VGG-16 transfer learning is proposed. In contrast to conventional methods, this study uses polynomial fitting to obtain the contours of a fishing vessel and transforms one-dimensional vessel contours into two-dimensional time-series images using the MTF coding method. The VGG-16 model is used for the recognition process, and migration learning is applied to improve the results. The UCR time-series public dataset is used as a transfer learning dataset for the MTF time-series image encoding. The experiment demonstrates that the proposed method exhibits higher accuracy and performance than 1D-CNN and other general neural network models, and the highest accuracy rate is 98.92%.

## Introduction

The automatic detection of vessel targets can improve the monitoring of vessels in rivers and seas through manpower, which can reduce the workload and management costs. Using a vessel identification system, illegal vessels can be effectively detected, and illegal acts can be recognized through the type and identification of the vessels. In the field of fishing vessels and commercial vessel management, vessel target detection is helpful for improving the level of sea area management and conserving marine resources^1^. Furthermore, in the military field, vessel target detection can aid in defense against enemies or can be used to gain an advantage in battles.

Small ships generally have a displacement of 50 tons or less, and are the main fishing vessels in offshore and inland rivers worldwide. With the rapid development of shipping and fisheries, waterway and fishing ground management are facing significant challenges. The management and monitoring of small fishing vessels require urgent informationization and automation. To date, the main techniques of vessel target detection systems have been based on radar scanning, automatic identification systems (AISs), or optical imaging, each of which exhibits several deficiencies.

The radar system can actively detect vessels by sending electromagnetic waves; it has all-weather and all-day detection capabilities. However, small vessels always have smaller reflection cross-sections and lower ship heights, and thus, small vessels are usually undetectable by conventional radar. The AIS is an automatic tracking system that uses transceivers on ships. It provides ship information such as the identification, position, course, and speed. The system requires ships to share information. This does not apply to noncooperative targets^2^. Optical imaging systems mainly contain visible-light CCTV and infrared systems. Owing to reflections in water or frequent rain and fog scenes, the collected images frequently exhibit interference. Therefore, this study proposes a method based on an infrared laser sensor to classify and identify hull contour structures from different vessels. This offers an effective method based on a different data model, which aids in improving the robustness of the entire vessel detection system.

**Figure 1:**
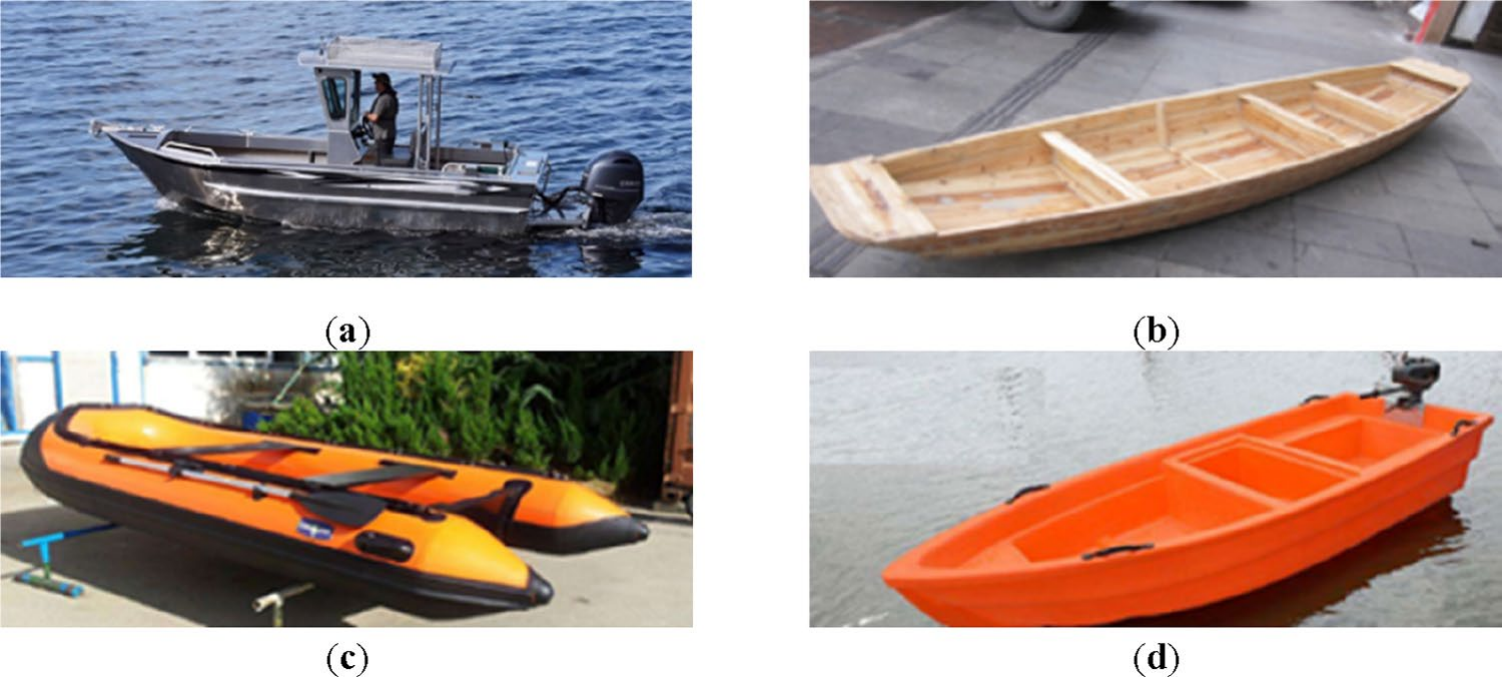
Types of four different types of small fishing vessels. (**a**) Small alloy fishing vessels, (**b**) wooden fishing vessels, (**c**) rubber inflatable fishing vessels, (**d**) PE plastic fishing vessels.

Laser sensors have been used extensively in obstacle detection and recognition, environment reconstruction, and the recognition of unmanned vehicles or ground mobile robots^3^. With the increasing research on and improvement of deep neural network algorithms, applications can reach a state-of-the-art level based on laser data^4,5^. Therefore, this study attempts to use a deep learning model to recognize small vessels based on a laser sensor. As illustrated in Fig. 1, different ships always have different sizes, shapes, and materials. All of these cause the laser point of the sequence to have different shapes and distributions. The method of encoding contour data can further expand the features of these contour data dimensionally, which is more conducive to the comprehensive capture of features by the deep learning network model and improve the identification performance of small fishing boats. Therefore, this study proposes a recognition method for small fishing vessels based on the Markov transition field (MTF) timing diagram and VGG-16 transfer learning.

In this study, paper proposes a new efficient method to recognize small vessels based on one dimensional signal of laser sensors. This is an additional method of multi model data application in small vessels recognition area. Meanwhile considering that the sample data is small, we train the model by using transfer learning based on the UCR time series public dataset. The one-dimensional laser signal is transformed into MTF images to expand the scale and dimension of signals. Then the different type of vessels will be easier to distinguish^6^. So, another contribution of this paper is that a modification block is proposed based on VGG-16 for improving the classification of MTF images.

Related work on vessel recognition can be divided into two main directions: conventional machine learning methods to realize vessel recognition and vessel recognition based on deep learning methods. Xia et al.^7^ proposed a vessel target detection algorithm based on a multifeature and variance-feature dynamic fusion model for optical remote sensing images. The vessel target was trained and predicted using a support vector machine based on the geometrical features. Damastuti et al.^8^ tested a real-time AIS database using KNN and neighborhood component analysis. The experiments classified ships in Indonesian waters based on the tonnage, length, and width. With the aim of the reliable and timely recognition of vessel targets in maritime battlefields, Guo et al.^9^ proposed a vessel recognition and distinguishing method based on the entropy of optical remote sensing data. According to the entropy, a decision tree based on hierarchical discriminant regression was constructed to identify different vessels in the data from an optical remote sensing system.

In contrast to simple machine learning research methods, several researchers have combined conventional algorithms with machine learning algorithms to enhance the advantages and improve the target recognition performance. Han et al.^10^ proposed a hierarchical process target recognition method based on evidence fractal analysis to solve the problem of incomplete images. Zhu^11^ and others presented a vessel detection method based on the shape and texture features for vessel optical image recognition. A new semi-supervised hierarchical classification method was used to distinguish vessels from non-vessels and the majority of false positives were eliminated. Khan^12^ proposed a vessel recognition method based on a directional gradient histogram and bag-of-words for infrared vessel images, and experimentally demonstrated the advantages thereof over other algorithms. Moreover, various researchers have used different algorithms to construct good vessel recognition models and have achieved good results. Zhang et al.^13^ introduced a vessel recognition method based on Bayesian reasoning and evidence theory and verified the proposed method in simulated combat scenes. The results indicated that, compared to other vessel recognition methods, the proposed method achieved performance advantages in terms of the recognition accuracy. Wang et al.^14^ proposed a support vector regression recognition method based on an improved particle swarm optimization algorithm to solve the problem of inaccurate models. In addition to machine learning technology, deep learning has rapidly developed, and many deep learning methods have been applied to vessel images to realize target recognition. Liu et al.^15^ developed an enhanced convolutional neural network (CNN) to improve vessel detection under different weather conditions. Chen et al.^16^ proposed a new vessel-type recognition framework based on deep learning, known as coarse-to-fine cascade CNN, and the model performance was proven experimentally. Huang et al.^17^ proposed a vessel detection method based on deep learning to solve the problem of detecting vessels of different sizes and types under complex sea conditions and improved the convolutional network. With the improvement in the resolution of synthetic aperture radar (SAR) images, Dong et al.^18^ presented a vessel classification framework based on a depth residual network for high-resolution SAR images. Lang et al.^19^ proposed an infrared intrusion target detection and classification method based on a neural network according to the characteristics of infrared target vessels and the detection difficulty. Ma et al.^20^ proposed a new concept that involves an improved YOLO v3 and KCF algorithm to obtain accurate recognition and authenticity detection of water targets.

Deep learning methods usually exhibit better accuracy and efficiency but often require large amounts of labeled data and high computational costs. In view of the problems of an insufficient labeled dataset, an unoptimized polarization image, and noise interference in vessel classification. Jeon et al.^21^ proposed a method that combined CNN and KNN models to improve the classification efficiency of vessels. To overcome the small number of datasets, Mishra et al.^22^ conducted a study on the transfer learning method in CNNs and tested it on the AlexNet, VGGNet, and ResNet architectures for ship classification tasks on MARVEL datasets. Li et al.^23^ presented a vessel recognition method based on a ResNet neural network and migration learning.

This study focuses on a laser sensor that outputs the one-dimensional contour data of vessels for the classification and identification of small fishing vessels. The fitting results of a one-dimensional contour are encoded into two-dimensional time-series images to enlarge the feature differences in terms of the scales and dimensions. Transfer learning is introduced into the model to achieve better accuracy and a lower computational cost. In “Materials and methods”, the image recognition method for small fishing vessels is described in detail. “Experiment” presents the analysis of specific related experiments, including the settings of the experimental equipment environment, preparation of datasets, and analysis of the experimental results. “Conclusion” concludes the paper.

## Materials and methods

**Figure 2:**
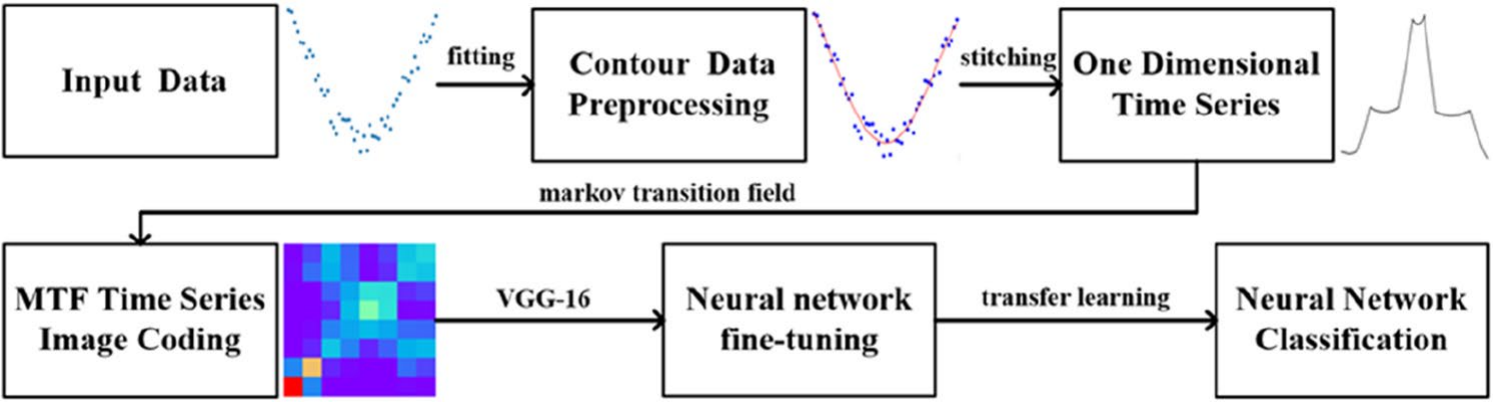
Flow chart of our method.

The contour data of different small fishing vessels are sampled using on the SICK laser contour sensor, following which the contour data are encoded into time-series images based on the Markov migration field^24^. The output images are input into the VGG neural network model for pretraining. Finally, pretrained parameter migration learning is applied to enhance the recognition effect of the neural network model. This study was conducted in three steps. The contour data of different fishing vessels were obtained using the SICK laser contour scanning equipment. The contour data were used as one-dimensional time series, which were encoded into two-dimensional time-series images. The classification and recognition of small fishing vessels were realized by VGG-16 neural network model migration learning, and the advanced nature of this method was verified experimentally. The flowchart of this study is shown in Fig. 2.

### Contour data fitting

**Figure 3:**
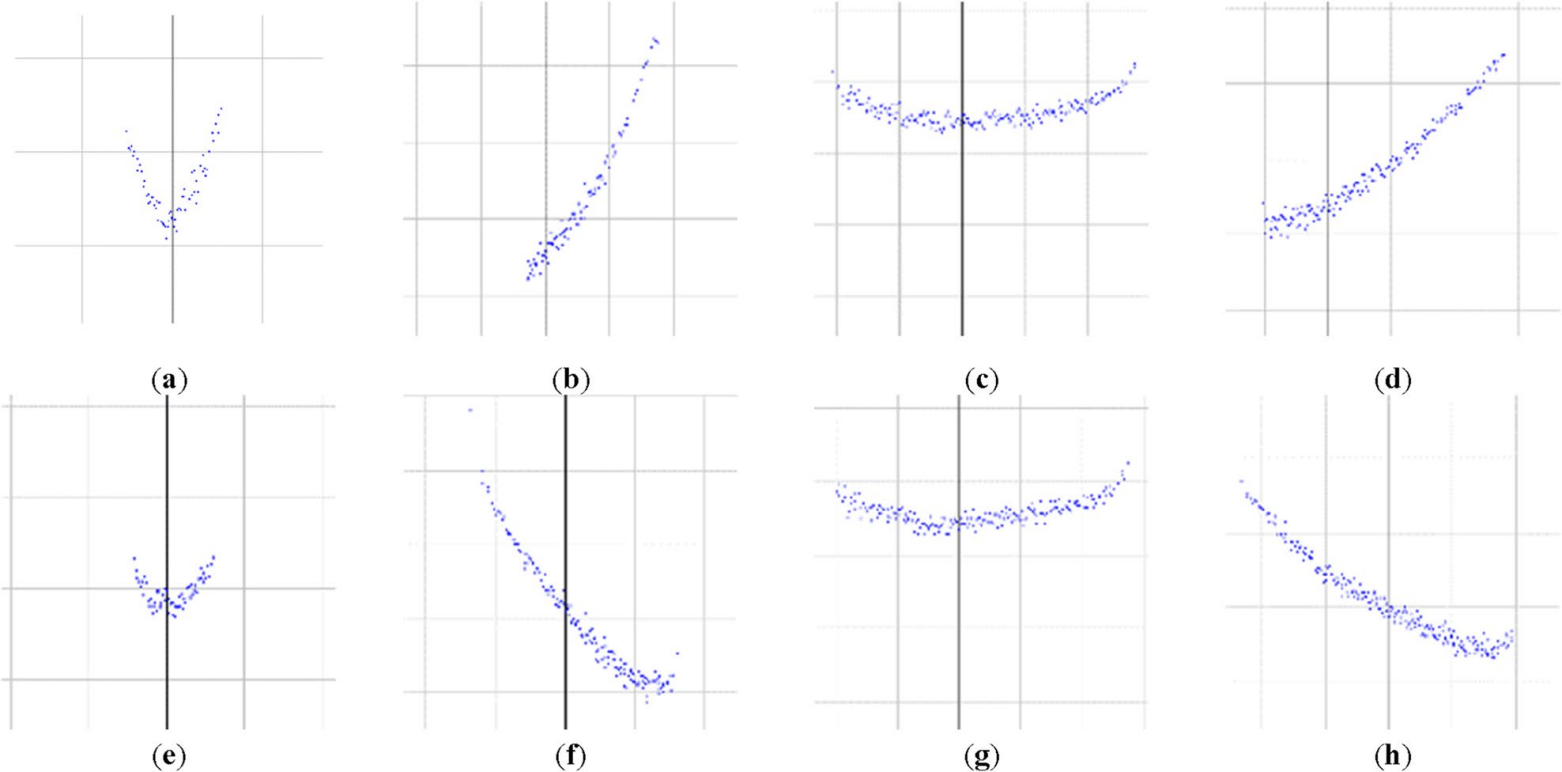
The scan results by LIDAR with different angle. (**a**) 0°, (**b**) 30°, (**c**) 90°, (**d**) 120°, (**e**) 180°, (**f**) 240°, (**g**) 270° scan result, (**h**) 300°.

As the contour data of fishing vessels are circling data from 0° to 360°, the contour data of fishing vessels from 0° to 360° are spliced into one-dimensional contour data according to the circling direction to retain the data characteristics of different fishing vessels to the greatest extent. The contour data are input as a one-dimensional time series of the Markov migration field. The profile data pattern of the fishing vessels sampled using laser sensing equipment is depicted in Fig. 3 (using a PE plastic fishing vessel as an example).

As the original vessel contour data are scattered, substantial invalid information exists; thus, it is necessary to fit and clean the data. Prior to fitting, to ensure the accuracy of the fitting results, the single scattered points far from the contour are eliminated. The cleaned vessel contour data are fitted using a polynomial curve fitting algorithm^25^, and the specific fitting process is described in detail below.

A polynomial curve fitting method is adopted for the fitting of scatter data approaching the curve. For a set of data, the fitting polynomial of $A=\left\{\left({u}_{0},{v}_{0}\right),\left({u}_{1}.{v}_{1}\right), \cdots \left({u}_{k-1},{v}_{k-1}\right)\right\},k\in {\mathrm{N}}^{*}$:

1 $$u={c}_{0}+{c}_{1}v+\cdots +{c}_{m}{v}^{m},m\in N$$

The sum of squares of errors is expressed as:

2 $${R}^{2}=\sum_{i=1}^{k}{\left[{y}_{i}-\left({c}_{0}+{c}_{1}{v}_{i}+\cdots +{c}_{m}{v}_{i}^{m}\right)\right]}^{2}$$

To obtain the qualified value, the partial derivative of the equation is obtained, and matrix (3) is obtained following simplification using the Vandermond matrix:

3 $$\left[\begin{array}{l}\\ 1 {v}_{0}\cdot \cdot \cdot {v}_{k-1}^{m}\\ 1 {v}_{1}\cdot \cdot \cdot {v}_{k-1}^{m}\\ \vdots \ddots \vdots \vdots \\ 1 {v}_{k}\cdot \cdot \cdot {v}_{k-1}^{m}\end{array} \right]\left[\begin{array}{l}{c}_{0}\\ {c}_{1}\\ \vdots \\ {c}_{k-1}\end{array}\right]=\left[\begin{array}{l}{u}_{0}\\ {u}_{1}\\ \vdots \\ {u}_{k-1}\end{array}\right]$$

Equation (3) can be abbreviated as Eq. (4):

4 $$V*C=U$$

**Figure 4:**
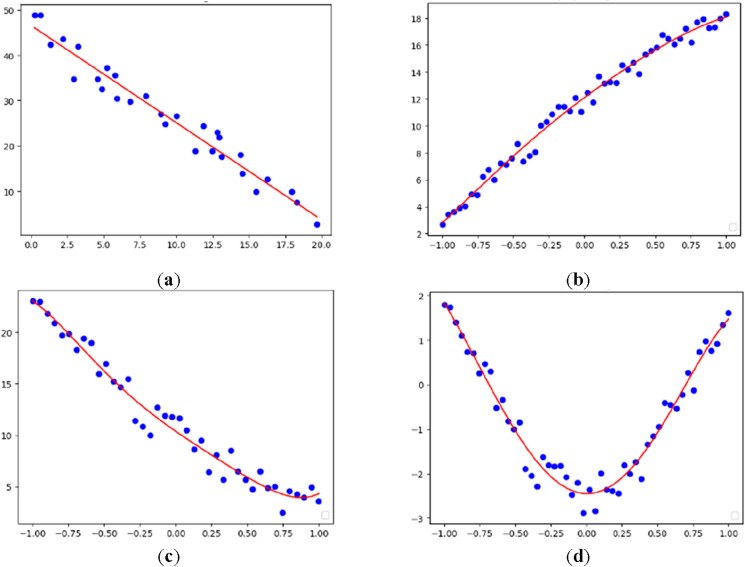
Parts of four types small fishing vessels data. (**a**) Small alloy fishing vessels, (**b**) wooden fishing vessels, (**c**) rubber inflatable fishing vessels, (**d**) PE plastic fishing vessels.

Equation (3) can be abbreviated as Eq. (4): where $V,$ $C,\mathrm{ and}$ $U$ correspond to the three matrices in Eq. (3) and the coefficient matrix is the required fitting curve result. After fitting the original contour data using the fitting algorithm, all of the fitting results are obtained, as indicated in Fig. 4.

### Contour data splicing

**Figure 5:**
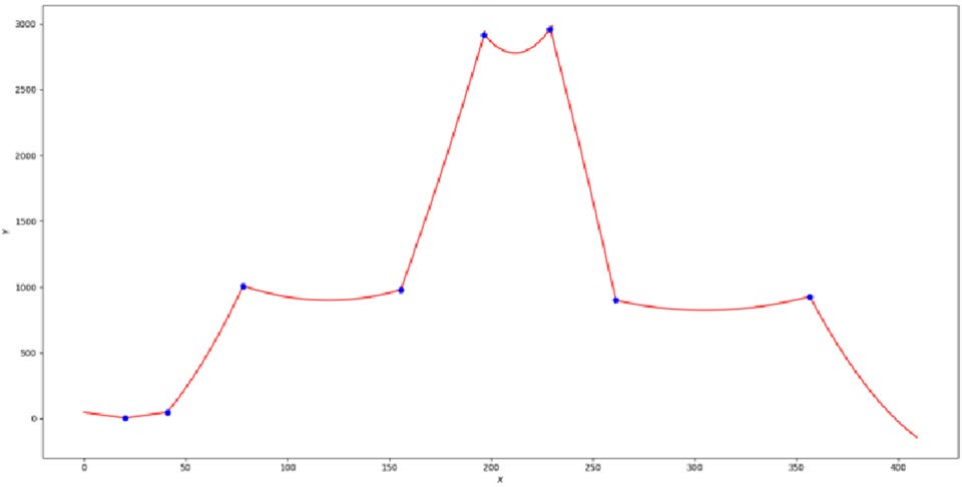
A renderings of all fitting results with the intersection point of fitting function equations as the splicing point.

After fitting all data of the fishing vessel contour, the fitting results must be spliced to generate the one-dimensional time series that are required in the following method. The fitting results are stitched according to the sequence of actual fishing vessel contours. In the splicing process, the intersection points of the fitting result function equations are used as the connection point, and the fitting results of each part are spliced into the final results of the continuous fitting^26^. The specific connection effect is illustrated in Fig. 5.

Following the splicing of the final fitting results, the data near the intersection point can be cleaned so that every value on the X-axis has a unique correspondence with a value on the Y-axis. In this manner, the final fitting result can be approximately treated as a special one-dimensional signal; that is, the fitting result can be regarded as one-dimensional time-series data for the input in the following method^27^.

### Contour data splicing

In the transition process of certain factors of a system, the n-th result is only affected by the n-1 result; that is, it is only related to the state in the previous moment and is unrelated to the past state. The concept of state transition is introduced into the Markov analysis. Markov state transition refers to the transition of objective objects from one state to another. The Markov transfer matrix is insensitive to the time dependence of the sequence; therefore, the MTF is introduced based on a first-order Markov chain and considering the time position relationship^28^. The specific connection effect is depicted in Fig. 5.

5 $$M=\left(\begin{array}{l} \\ {m}_{\mathit{ij}}|{t}_{1}\in {u}_{i},{t}_{1}\in {u}_{j} \cdot \cdot \cdot {m}_{\mathit{ij}}|{t}_{1}\in {u}_{i},{t}_{n}\in {u}_{j} \\ {m}_{\mathit{ij}}|{t}_{2}\in {u}_{i},{t}_{1}\in {u}_{j} \cdot \cdot \cdot {m}_{\mathit{ij}}|{t}_{2}\in {u}_{i},{t}_{n}\in {u}_{j} \\ \vdots \ddots \vdots \\ {m}_{\mathit{ij}}|{t}_{n}\in {u}_{i},{t}_{1}\in {u}_{j} \cdot \cdot \cdot {m}_{\mathit{ij}}|{t}_{n}\in u,{t}_{n}\in {u}_{j}\end{array}\right)$$

**Figure 6:**
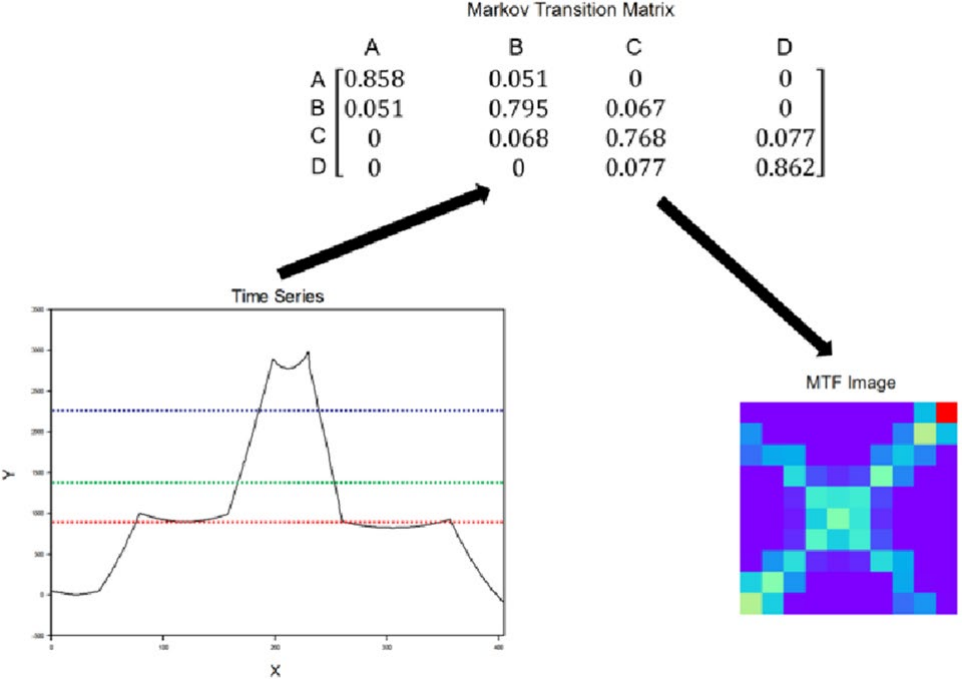
One-dimensional time series encoding MTF time series image process.

The one-dimensional contour data of the fishing vessels are regarded as one-dimensional time-series data, which are defined as a series $T=\{{t}_{1},{t}_{2},\dots ,{t}_{n}\}$. When each ${t}_{i}$ in the time series $N$ is assigned to its own storage box ${U}_{j}(j\in [1,U])$, the weighted adjacency matrix $M$ is transformed into $U\times U$ between the counting point boxes of the first-order Markov chain. To overcome the disadvantage that $m$ is insensitive to the time dependence of the $T$ distribution and time step size, the MTF is defined as follows:

In the MTF method, ${m}_{ij}$ refers to the ${u}_{i}\to {u}_{j}$ transition probability. Considering the time position, matrix $m$ including the transition probability is extended to the MTF matrix on the dimension axis. The principal diagonal in the MTF matrix is ${m}_{ij}$, which captures the probability of self-transition from each quantile to itself (self-transition probability). To improve the computational efficiency, a fuzzy check is used to average the pixels in each non-overlapping patch $m\times m$; that is, to aggregate the transformation probability in each subsequence with length $m$. The one-dimensional time-series data of the fishing vessels are encoded into MTF matrix images, as shown in Fig. 6.

### VGG-16 neural network model

CNNs have been used extensively in computer vision recognition tasks in recent years. Neural networks have achieved state-of-the-art results compared to conventional methods, and image classification is an important task in vision recognition. However, shallow neural network models exhibit certain limitations in large-scale image recognition tasks.

The VGG-16 neural network model is proposed to explore the performance of deeper network models further. The VGG-16 neural network uses an architecture with an extremely small (3 × 3) convolutional filter and the depth of the network structure is increased. The network performance is significantly improved by increasing the depth to 16 to 19 weight layers^29^.

The network uses 13 convolutional layers and three fully connected layers. The core size of the convolutional layer in the VGG-16 network architecture is 3 × 3 and the step size is 1. The pooled layers are all 2 × 2 with a step size of 2. The input size of the network is 224 × 224 pixels. Following each pooling, the size of the feature map is reduced by half. In the last characteristic diagram of the network, the architecture before the fully connected layer is 7 × 7 × 512 channels. This layer is expanded to 25,088 channels. The final layer of the network is the authentication probability of softmax layer. All images in the VGG-16 network are of the same size; therefore, this network is also one of the most effective methods for applying the transfer learning algorithm.

ReLU is applied to the model because it is known to transmit errors better than the sigmoid function^30^. The VGG-16 network uses a 224 × 224 pixel image as the default input image. The ReLU activation function is applied to the three fully connected layers after the VGG-16 convolutional layer. A hidden layer is added after each layer, and the dropout layer is inserted, the value of which is set to 0.2. This can cause the hidden layer to prohibit the use of 20% nodes randomly, thereby avoiding overfitting^31^. The activation function for the output layer is the classic softmax classifier.

### Transfer learning of neural network

It is very unusual to train CNNs from scratch because the training of neural networks generally requires numerous training samples and a powerful GPU for deep learning. In the method based on transfer learning, the optimal pretraining model weights are trained for similar problems to be used for new image recognition tasks. In transfer learning, any pretrained convolutional network can use millions of images as a fixed feature extractor. Thus, fine-tuning can be applied for specific datasets and problems, and the network weights can be trained in advance to achieve the best network training effect^32^. The comparison and analysis of the correlation between image type and size of data set in the transfer learning method are shown in Table 1.

**Table 1:** Comparative analysis of image type correlation and size correlation of data set in transfer learning method.

	Factor		Factor size	Input size
	Similarity	Size		
1	Similar	Similar	Only one classifier layer is trained using pre-trained weights	When the new data set is small, the fine-tuning of pre-training weights will lead to over-fitting. In the new problem, the image is similar to the original data set, and the features are still related through pre-trained weights
2	Similar	Larger	Overall network fine tuning	When the new data set is large enough, there will be no fitting problem in retraining
3	Dissimilar	Similar	A few layers can be fine-tuned	Because of the difference between the new data set and the original data set, few convolutional layers need to be retrained to learn features
4	Dissimilar	Larger	Transfer learning can fine-tune the whole network	The new data set is large enough and the image difference is large, so transfer learning can be utilized by fine-tuning the whole network

As the original VGG-16 neural network model was trained using ImageNet, the dataset contains 1000 image categories. However, in this study, the number of images of small vessels is smaller compared to the ImageNet dataset. In this study, the efficiency of the standard VGG-16 model, which is directly used as the classification and recognition model, needs to be improved. In view of the influence of different situations on the effect of transfer learning, we analyze the correlation between the new dataset and original ImageNet dataset type as well as the correlation between the dataset sizes, as indicated in Table 1. The analysis reveals that the small fishing vessel dataset in this study does not have a certain correlation with the original dataset; therefore, case 3 is considered in the transfer learning process.

## Experiment

### Experiment environment

#### Experiment settings

Owing to the need for a computer GPU in the training process of the VGG-16 neural network in deep learning, an Alienware R12 integrated machine equipped with an Intel i9 processor and an RTX 3090 graphics card was used as the running hardware environment of the neural network in our experiment.

Python, which is a high-level programming language, is frequently used by researchers in deep learning owing to its simple, open-source, portable, object-oriented, and rich third-party libraries. The experimental software environment in this study was also built using the PyCharm development tool based on the Python language, and the Python environment used the TensorFlow GPU 2.2 and Keras 2.2.4 framework.

#### Experiment dataset

**Figure 7:**
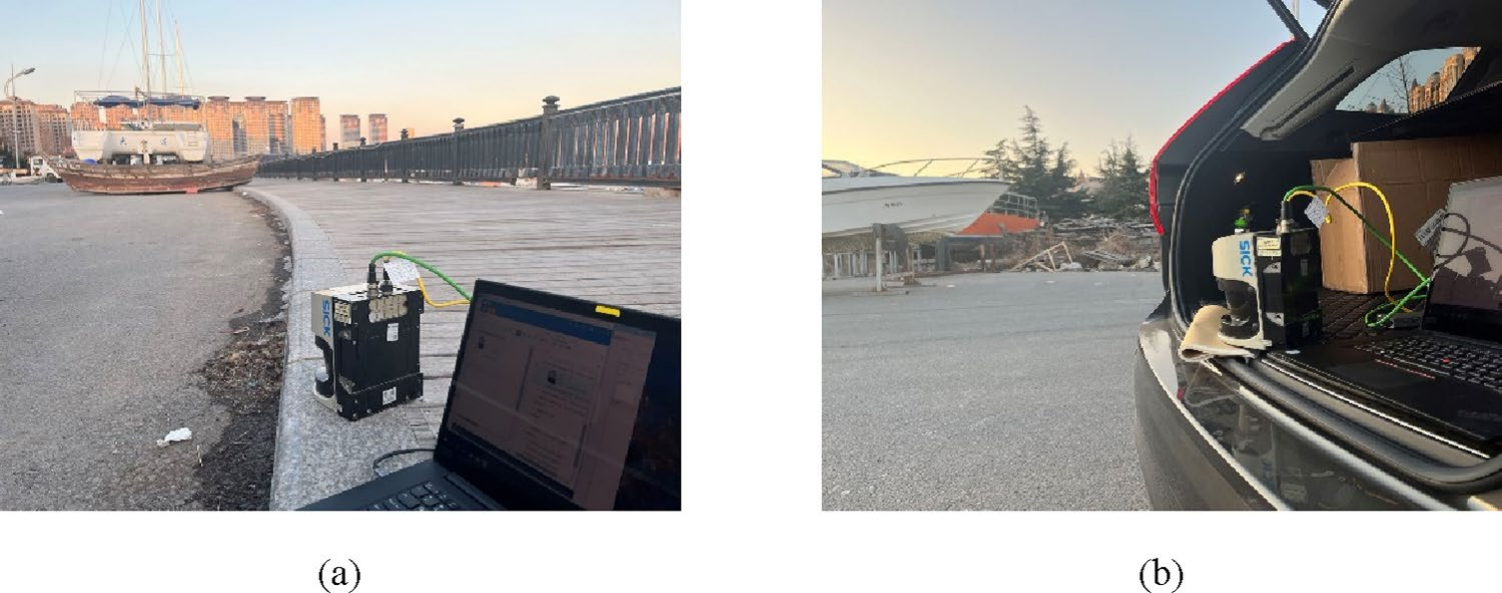
The data sampling of fishing vessel by SICK laser scanner, (**a**) wooden fishing vessels, (**b**) PE plastic fishing vessels.

The dataset is an essential part of the model training and verification processes. We used the LMS series infrared laser scanner equipment manufactured by SICK LMS511 to sample the dataset of local small fishing vessels. We focused on four different types of small fishing vessels: small alloy fishing vessels, wooden fishing vessels, rubber inflatable fishing vessels, and PE plastic fishing vessels. Following authorization, we used infrared laser sensors to sample the contours of these small fishing vessels using a 360-degree surrounding sampling method, the sampling process is as Fig. 7.

As a result of time required and the epidemic policy, a total of 3,268 different contour data were eventually sampled after approximately half a year of collection and sampling. The data samples of small alloy fishing vessels accounted for the highest proportion in all datasets, with 1063 sample data. Small wooden fishing vessels constituted 918 sample data. Furthermore, there were 726 sample data for PE plastic small fishing vessels. Finally, small inflatable rubber fishing vessels accounted for the least sample data at 561.

**Figure 8:**
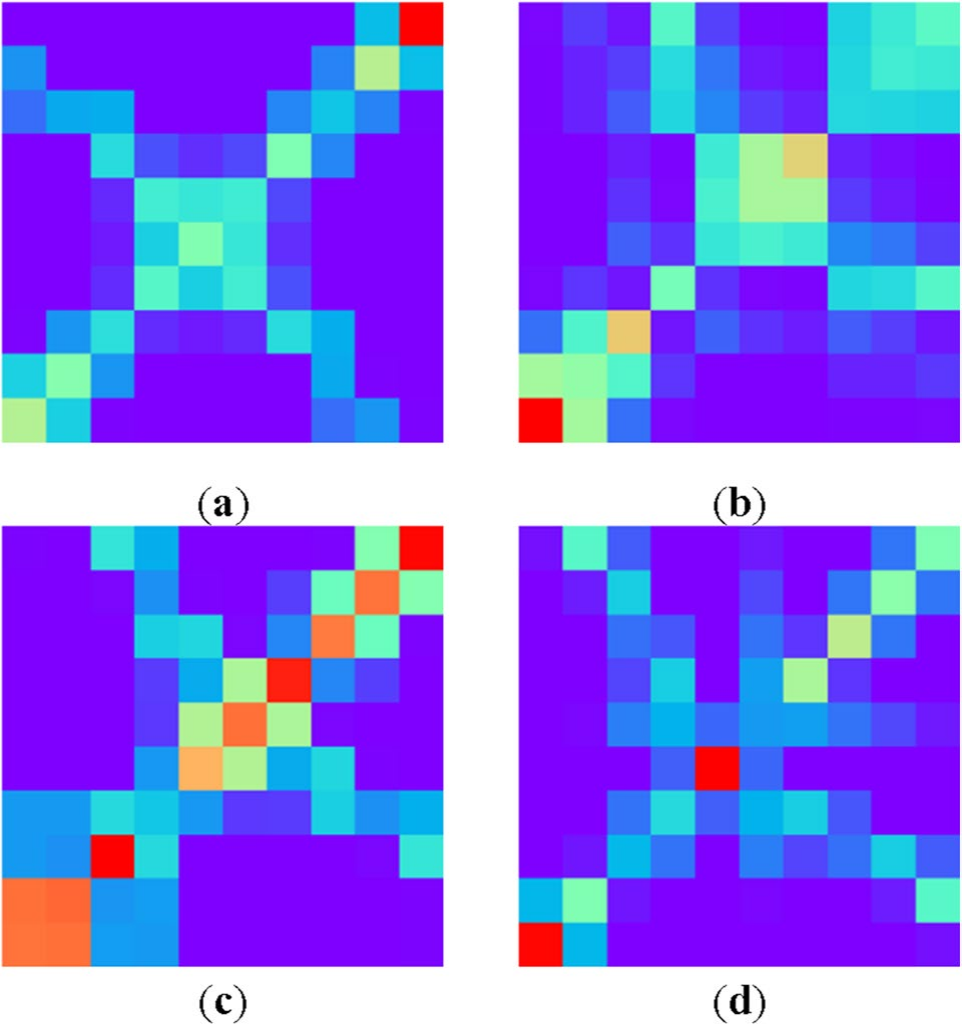
MTF two-dimensional time series images corresponding to four different types of small fishing vessels, (**a**) MTF images of small alloy fishing vessels, (**b**) MTF images of wooden fishing vessels, (**c**) MTF images of rubber inflatable fishing vessels, (**d**) MTF images of PE plastic fishing vessels.

We used the same fitting method to stitch the fitting results for all contour data to create, one-dimensional time-series data for use in the subsequent image coding. A consistent two-dimensional time-series image coding method (MTF) was adopted to encode all one-dimensional time series to generate reliable time-series image dataset samples. Four different types of MTF time-series images were generated, as illustrated in Fig. 8.

To improve the results, we enlarged our dataset of four types of small vessels using data augmentation, which included flipping, scaling, and noise addition. The vessel images were flipped horizontally. In the scaling process, the scale factors were 0.75 and 1.5. Overfitting generally occurs when a neural network learns high-frequency features, which may not be helpful to the task of the network and may affect the low-frequency features. To eliminate high-frequency features, once the data image expansion was completed, Gaussian noise and salt-and-pepper noise data were randomly added to the image samples to offset the influence of high-frequency image features^33^.

After completing all of the expansion steps, we cleaned the data samples of the expanded dataset and selectively removed several poor-quality image samples; thus, the number of samples for each image type was 3,600. The sample quantity distributions of all image datasets following the sample expansion are presented in Table 2.

**Table 2:** Distribution of original sample number of MTF time series images of small fishing vessels.

Type	Original	Number
Small alloy fishing vessels	1063	3600
Wooden fishing vessels	918	3600
Rubber inflatable fishing vessels	726	3600
PE plastic fishing vessels	561	3600
Total	3268	14,400

### Experimental process

#### Data enhancement and setup

The sample size has a significant influence on the learning ability of the neural network. To enable the neural network to learn more sample features, we expanded the number of time-series images of various types of samples. Dataset expansion methods such as rotation, scaling, and translation were adopted to obtain as many types of dataset samples as possible.

To reduce the model processing complexity of the model, the distribution of the datasets was balanced in the preparation stage, and all dataset samples following enhancement and expansion are presented in Table 4. The overall dataset consisted of four different types of small vessels, with a total of 14,400 MTF time-series images, including 3,600 images of small alloy fishing vessels, wooden fishing vessels, rubber inflatable fishing vessels, and PE plastic fishing vessels. Figure 8 depicts representative images of each fishing vessel category.

The complete dataset was divided into a 3:1 ratio distribution that was used for training and testing, respectively. One-third of the training datasets were used for verification. The image generated by the MTF method was a pseudo-color image. In the experimental encoding, we performed color mapping of the image according to each value in the Markov matrix such that the MTF image became a color time-series image with three RGB channels. Moreover, owing to the standard input of the image size of the neural network model, all images had to be unified to a size of 3 × 224 × 224.

#### Fine-tuning of VGG-16 model

VGG-16 was originally designed to extract image detail features from 1000 ImageNet datasets in the experimental training process. However, the higher-order features that are learned by the neural network model trained in this manner are not directly related to the categories of vessel MTF two-dimensional time-series images in this study, which would result in a poor training effect. Therefore, several convolution blocks were retrained, and the weights of the VGG-16 neural network were fine-tuned to achieve better recognition and classification results. To improve the results of the network model, we modified the original VGG-16 neural as follows:

- Parameter settings: we used the Adam optimizer to optimize the learning rate parameter during pretraining. Based on the experience of many experiments, the batch size was set to 32. The number of neural network model training iterations was set to 200, and batch normalization and dropout layers were added.
- Convolution retraining: first, the first three convolution blocks of the VGG-16 neural network model were frozen, and convolution blocks 4 and 5 were retained, so that the weights of these two convolution blocks in the VGG-16 neural network model were retrained using the dataset of small vessels. This made the neural network model more suitable for efficient vessel classification and recognition tasks.
- Additional convolution: while freezing the first three convolution blocks of VGG-16, we added a new module consisting of three convolutional layers and a maximum pooling layer after convolution block 5 and before the fully connected layer. The purpose of adding the new module was to combine the batch normalization and dropout to avoid overfitting of the neural network model, so that more vessel features could be learned.

**Figure 9:**
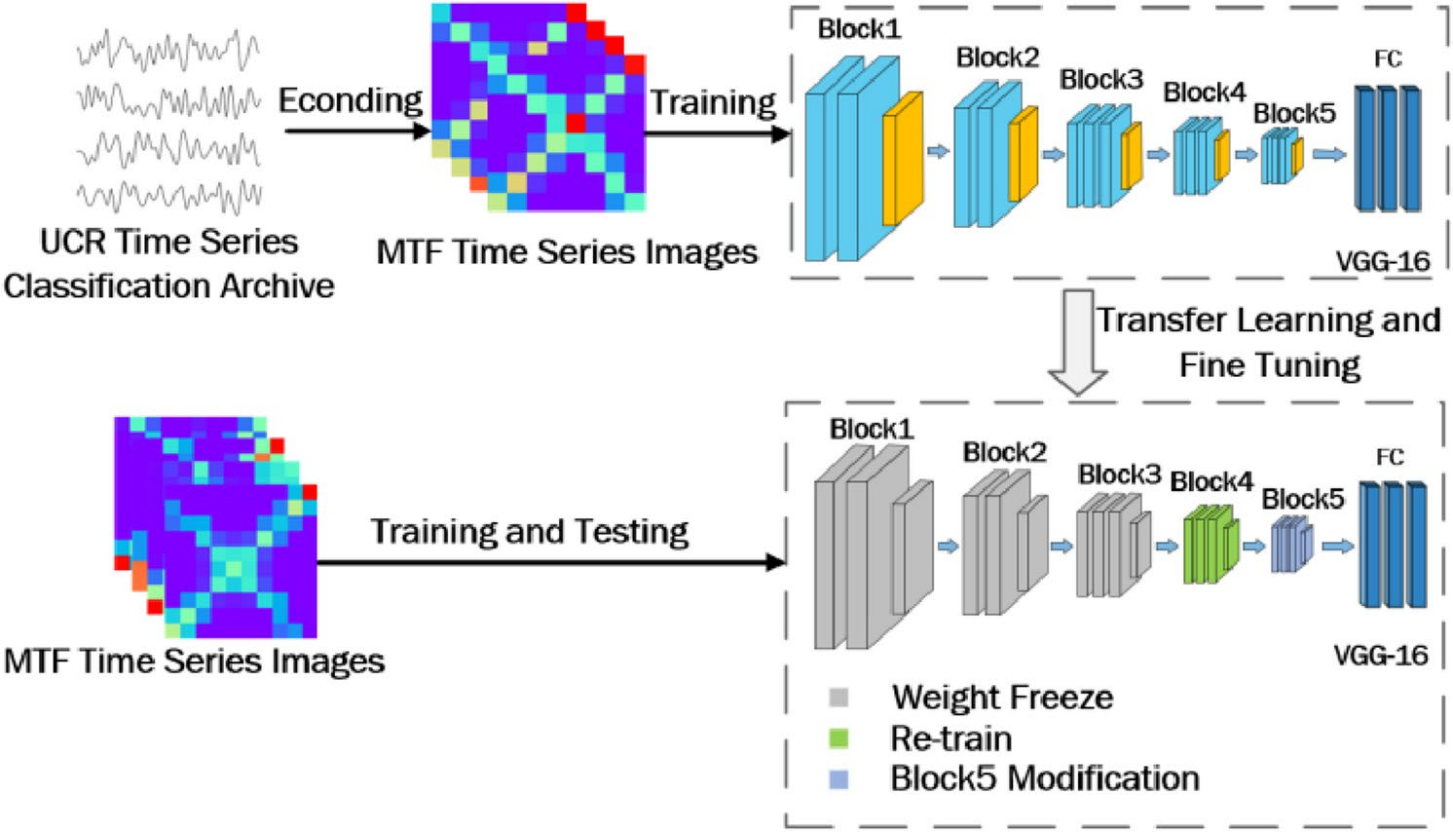
VGG-16 neural network model structure fine-tuning modification picture.

**Figure 10:**
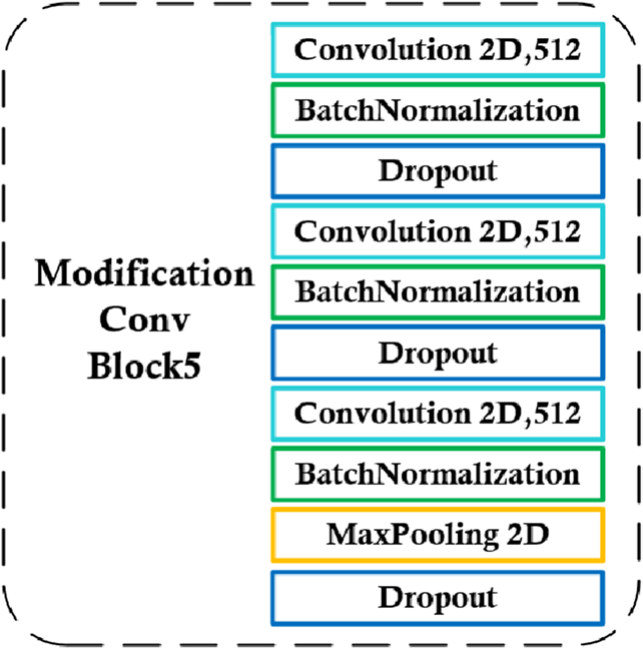
Modification of Conv Block-6 of VGG-16 neural network model.

Figure 9 presents a schematic of the overall fine-tuning of the VGG-16 model structure. The VGG-16 model was divided into five convolutional modules. During fine-tuning, because VGG-16 usually obtains common features in the first three convolutional modules, and more distinguishing features are located close to the fully connected layer, we froze the first three convolutional modules of the VGG-16 for training the weights of the neural network, leading to more resources in the last modules during training. Therefore, the training time and resource utilization efficiency of the network model could be greatly improved. For the network model to adapt to the small-ship identification task more effectively, we retrained convolutional modules 4 and 5. Meanwhile, we modified convolutional module 5 to improve the model efficiency. The batch normalization and dropout layers were newly added to convolutional module 5. The structure of the newly modified convolutional module is depicted in Fig. 10. The added batch normalization layer could accelerate the training speed and increase the learning rate, and even if the learning rate was low, it could achieve fast learning. Because our dataset was not sufficiently large, the neural network training was prone to overfitting. The new dropout layer was added to prevent overfitting, and to provide more stable and reliable network model training results.

### Transfer learning

The UCR time-series archive, which was introduced in 2002, has become an important resource in the time-series data mining community, with at least 1,000 published papers making use of at least one dataset from the archive. We used the full UCR dataset for the MTF encoding process and used the encoded MTF time-series images as the dataset for transfer learning. As the UCR dataset is a standard time-series archive, the use of MTF time-series image encoding was very suitable. The encoding of the full dataset with the same parameters would yield better results, thereby playing a strong role in the transfer learning process.

The fine-tuning of the VGG-16 neural network model leaves the full pretrained parameters for the subsequent learning process. In the transfer learning process, first, all of the two-dimensional time-series images of various types of small vessels generated by the MTF method were randomly disturbed; thereafter, 7500 images of the 14,400 images in the dataset were used as training sets, 2500 images were used as test sets, and 2500 images were selected as verification sets.

After several rounds of training and learning in the training process, we obtained more suitable parameters for the classification and recognition of small vessel datasets, such as the iteration times and batch size parameters, and identified a better Adam optimizer to prevent network overfitting. As indicated in Table 3, under the network parameters of an initial learning rate of 0.001, batch of 32, momentum of 0.95, and 200 iteration times, the average highest accuracy rate of small fishing vessel recognition and classification was 98.92%. In the following section, a comparison between the proposed method and conventional neural network models is presented.

**Table 3:** Setting of super parameters of neural network during experiment.

Experimental parameters	Number of testing
Learning rate	0.001
Batchsize	32
Momentum	0.95
Number of epochs	200
Dropout	0.3–0.5
Optimizer	Adam

### Analysis and results

We compared the proposed method with other conventional network models; the classification performance index information is presented in Table 4. The difference between the training and verification accuracies of the conventional VGG-16 model was 3.84%. In the proposed fine-tuning model for transfer learning, the gap between the training accuracy of the VGG-16 model after transfer learning and that before the test was obviously reduced, becoming 1.06%, which indicates that overfitting in the network was reduced and the convergence of the network model was improved. In other aspects, the overall accuracy of the VGG-16 network model after transfer learning was obviously improved by 6.79% compared to the overall accuracy of the conventional VGG-16 network model.

Moreover, compared to the conventional network models, the experimental method exhibited a greater performance improvement. Compared to the 1D-CNN network model, which directly uses the outline data of small fishing vessels, the overall accuracy improvement was the greatest, with a difference of 13.98%. ResNet-50, which is a neural network model with higher and deeper levels, had the smallest gap, with a difference of only 1.35%; however, the large network model requires a larger GPU and longer training time. As can be observed from Table 4, the training time of the ResNet-50 neural network was the longest, reaching 5 h and 17 min. In contrast, the 1D-CNN neural network had the shortest training time at 1 h and 39 min, but it lacked accuracy. It can be observed from the table that our proposed method was the most cost-effective in terms of time and accuracy.

**Table 4:** Comparison of the overall accuracy between the proposed method model and other neural network models in this table.

Model	Training (%)	Testing (%)	Tenfold CV (%)	Time
1D-CNN^35^	87.12	84.93	83.83	1 h 17 min
AlexNet^36^	91.54	88.19	88.01	1 h 52 min
VGG-16^37^	95.96	92.12	91.83	2 h 56 min
VGG-19^38^	97.21	94.78	93.98	3 h 29 min
ResNet-50^39^	98.67	97.56	97.29	3 h 40 min
The Proposed	99.97	98.92	98.44	3 h 22 min

**Figure 11:**
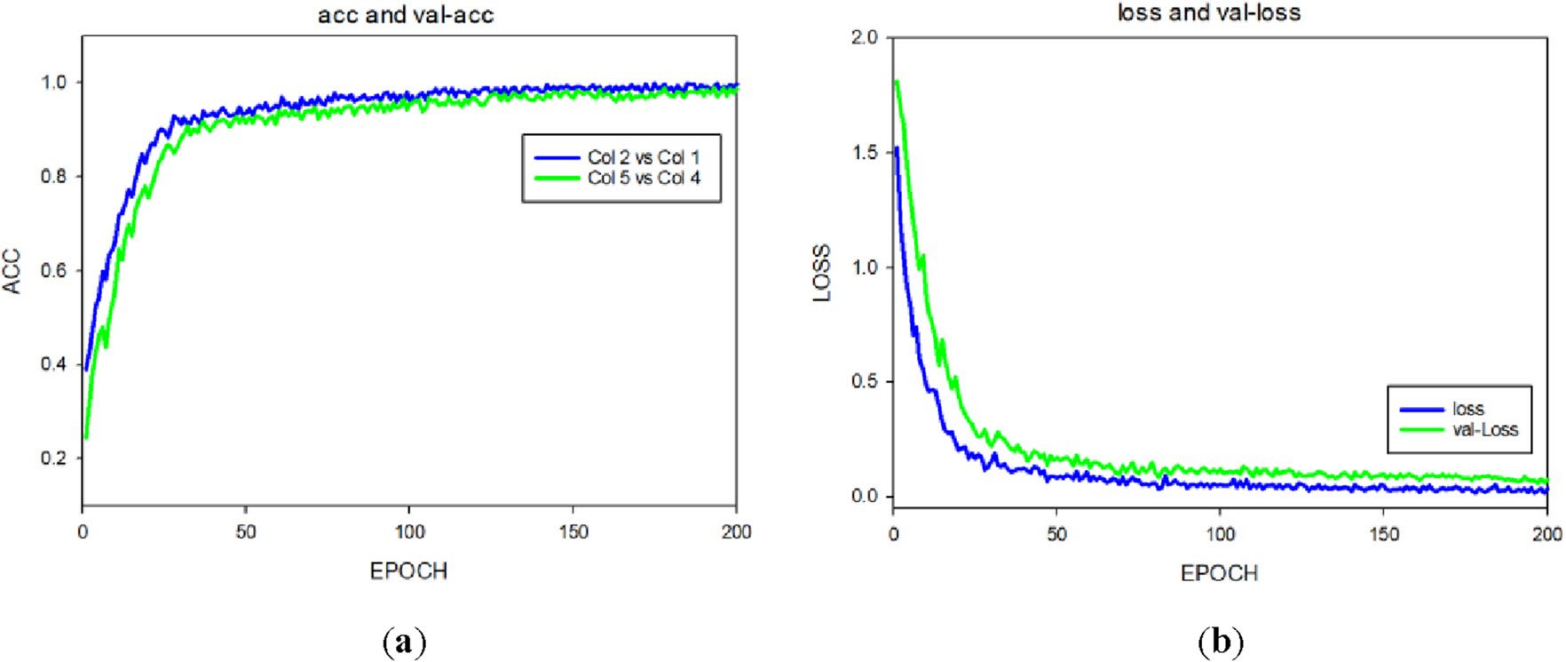
Model training curve results. (**a**) Classification accuracy graph, (**b**) loss graph.

Figure 11 presents the training image of the proposed model, which exhibited better performance and convergence. The figure shows that the training curve of the model was stable overall, including the training accuracy, test accuracy, and loss curves. After fine-tuning the VGG-16 model, the curve fluctuation of the training and test accuracies of the training curve in the transfer learning process was relatively small, the overall training process was relatively stable, and no jitter, overfitting, or underfitting occurred in the entire process^34^.

In this study, the contour data of small fishing boats were fitted to a spliced contour data sequence to encode time-series images. The application of transfer learning technology and network structure adjustment in the model was proposed. We used the MTF method to encode the contour data of small fishing boats, and skillfully transferred the distribution characteristics of the data points of different contours around fishing boats to two-dimensional time-series images to transform as many contour features of the fishing boats as possible into two-dimensional image inputs that are more sensitive to neural networks. Moreover, the transfer learning technology and overall structure of the VGG-16 neural network were fine-tuned. By freezing the weights of the convolutional modules, retraining, and adding new convolutional modules, the model exhibited a higher initial performance, better improvement rate, and more stable convergence effect. In the experiment, we used a tenfold cross-validation (CV) model validation method to verify all neural network models. The final results are presented in Tables 4 and 5. The results demonstrate that our method had a more stable performance and better effect than the other five neural network methods.

**Table 5:** The classification performance index matrix of the model.

Model	Precision (%)	Recall (%)	F1 (%)	Accuracy (%)
1D-CNN	84.94	84.93	84.94	84.93
AlexNet	88.19	89.68	88.93	88.19
VGG-16	92.12	92.20	92.16	92.12
VGG-19	94.79	94.83	94.81	94.78
ResNet-50	97.61	97.57	97.59	97.56
The Proposed	99.16	98.92	99.04	98.44

Tables 4 and 5 summarizes that the classification performance indices of the proposed model were all higher than those of the other models. From Table 4, we can see that although our method is not the fastest in Mo model reasoning time, it has good reasoning time cost-performance while maintaining high accuracy performance. Compared with the original VGG-16 model, although our method took more time in the process of transfer training, it significantly improved the performance of the model, with a significant improvement effect of 6.8%. In addition, the time of the proposed method is less than that of ResNet-50 and VGG-16 networks, but the accuracy of the proposed method is still higher than that of the proposed method.

From Table 5, The precision index of the proposed model was the highest (99.16%), and the tenfold CV index of the 1D-CNN model was the lowest (83.83%). Compared to the 1D-CNN model with a simple structure, with an increase in the complexity of the neural network model, the accuracy index was improved by 13.99% and the tenfold CV index was improved by 14.61% from the AlexNet neural network model to our transfer learning model method. Furthermore, compared to the original VGG-16 neural network model based on non-transfer learning, the model using the proposed transfer learning technique also exhibited a significant performance improvement, with a 6.8% improvement in the accuracy index and 6.61% improvement in the tenfold CV index. The ResNet-50 neural network exhibited several advantages in the indices and was closest to the performance of the proposed method, but it requires considerable calculation and time costs.

## Conclusion

In this study, four different types of small fishing vessels were investigated, and a small fishing vessel recognition method based on MTF time-series images and VGG-16 migration learning was proposed to solve the problem of small fishing vessel classification. In contrast to conventional practice, a new processing method for fitting the scatter data of small fishing vessel contours based on an infrared laser sensor was presented. The fitting function equations were gradually spliced into one-dimensional time-series data and encoded into two-dimensional time-series images. Finally, the VGG-16 neural network model was used for migration learning, and the neural network classification and recognition model were fine-tuned and improved during the experimental process.

The final experimental results demonstrate the effectiveness of the proposed method compared to the 1D-CNN and conventional neural network models. Four types of vessels were evaluated and analyzed using limited datasets. The experiments revealed that the method had the highest average accuracy of 98.92% for the four types of vessel datasets.

## Data Availability

The datasets generated during the current study are not publicly available due [the dataset is a work in progress] but are available from the corresponding author on reasonable request.
